# Gestational Diabetes Mellitus: A Positive Predictor of Type 2 Diabetes?

**DOI:** 10.1155/2012/721653

**Published:** 2012-05-23

**Authors:** Gregory E. Rice, Sebastian E. Illanes, Murray D. Mitchell

**Affiliations:** ^1^The University of Queensland Centre for Clinical Research, RBWH Campus, Herston, Brisbane, QLD 4029, Australia; ^2^Department of Obstetrics and Gynecology, Universidad de Los Andes, San Carlos de Apoquindo 2200, Las Condes, Santiago, Chile

## Abstract

The aim of this paper is to consider the relative benefits of screening for type two diabetes mellitus in women with a previous pregnancy complicated by gestational diabetes mellitus. Recent studies suggest that women who experience GDM are at a greater risk of developing type 2 diabetes within 10–20 years of their index pregnancy. If considered as a stand-alone indicator of the risk of developing type 2 diabetes, GDM is a poor diagnostic test. Most women do not develop GDM during pregnancy and of those that do most do not develop type 2 diabetes. There is, however, a clear need for better early detection of predisposition to disease and/or disease onset to significantly impact on this global pandemic. The putative benefits of multivariate approaches and first trimester and preconception screening to increase the sensitivity of risk assignment modalities for type 2 diabetes are proposed.

## 1. Introduction

The keystone to improving disease management and health outcomes remains the early and accurate diagnosis of the predisposition to, or onset of, disease. Early detection of disease risk and onset is the first step in implementing efficacious treatment and improving patient outcomes. In the context of screening for prediabetic and diabetic conditions in asymptomatic individuals, early detection may allow the implementation of dietary, lifestyle, and/or pharmacologic interventions that limit or prevent the development of disease-specific pathophysiologies. The rationale for seeking to develop predictive tests for diabetes and other metabolic disorders, thus, is clearly evident.

Recent studies suggest that women who experience gestational diabetes mellitus (GDM) are at a greater risk of developing type 2 diabetes mellitus (type 2 diabetes) within 10–20 years of their index pregnancy [1]. Monitoring glycemic control and intervention strategies to delay or prevent disease onset have been advocated in such women. Type 2 diabetes, however, is a disease of heterogeneous aetiology and GDM is but one risk factor. If considered as a stand-alone indicator of the risk of developing type 2 diabetes, GDM is a poor diagnostic test. Most women do not develop GDM during pregnancy and of those that do most do not develop type 2 diabetes. Postpartum monitoring of women who developed GDM during pregnancy, nevertheless, may be of clinical utility in this higher risk cohort. There is, however, a clear need for better early detection of predisposition to disease and/or disease onset to significantly impact on this global pandemic.

For women (and their partners), pregnancy represents a period of increased interaction with the healthcare system and a period where changes in lifestyle may have significant impact not only on the parents but also on the disease susceptibility of the next generation. This period represents an opportunity to implement more comprehensive educational, lifestyle, and disease susceptibility or onset screening initiatives, either during first trimester or, perhaps more effectively, in the setting of a preconception clinic. The objectives of preconception care are to promote the health of women (and their partners) before conception and thereby improve pregnancy-related outcomes (both current and future) and to reduce the risk of adult-onset disease (e.g., cardiovascular and metabolic) in their children. Over the next decade, the combined effects of an increase in the incidence of type 2 diabetes in younger women [2] and an increased maternal age at first delivery will result in an increased number of pregnancies exposed to the potentially adverse effects of undiagnosed diabetic conditions. Globally, the number of live births each year is estimated to be more than 134 million. Preconception programs, thus, have the potential to specifically target more than 360 million individuals per year (i.e., the parents and the offspring).

The aim of this paper is to consider the relative benefits of screening for type 2 diabetes in women with a previous pregnancy complicated by GDM. The putative benefits of multivariate approaches and preconception screening to increase the sensitivity of risk assignment modalities for type 2 diabetes are proposed.

## 2. Diabetes Mellitus

### 2.1. Epidemiology

Diabetes mellitus is one of the most common chronic diseases. The number of adults with diabetes has more than doubled over the past 30 years. Recent reports by Danaei et al. [3] and Shaw et al. [4] estimate the global prevalence of diabetes to be between 285 and 347 million people. By 2030, diabetes is expected to affect 552 million people. The prevalence of this disease is increasing in most countries as changing lifestyles lead to reduced physical activity and increased obesity [4]. Since 1980, the age-standardised fasting plasma glucose concentration has increased by 0.07 mmol/L per decade for men and 0.09 mmol/L per decade for women [3]. These data are consistent with an overall population-based decline in glycemic control.

The incidence of diabetes has also increased dramatically in women of reproductive age (i.e., 18*–*44 years). For example, in the USA, the rate of diabetes has increased by 70% in women aged 30–39 years over the past decade. It is estimated that 12.6 million women (or 10.8% of women over the age of 20) have diabetes, and of these women, 90–95% have type 2 diabetes. Death rates for women aged 25–44 years with diabetes are more than 3 times that for women without diabetes [5].

### 2.2. Aetiology of Type 2 Diabetes

The available data are consistent with type 2 diabetes being of genetic origin; however, its precise aetiology remains to be unequivocally established. Behavioural, lifestyle, and environmental factors have all been implicated as modifiers of disease risk. Recent genome-wide screening studies have identified multiple susceptibility variants consistent with type 2 diabetes being of polygenic origin [6–10]. Thus, type 2 diabetes may be a phenotypic manifestation of many different aetiologies that simply share hyperglycemia as a common outcome [11].

A hallmark of the onset of type 2 diabetes is a progressive decrease in insulin-stimulated glucose uptake. High circulating concentrations of glucose induce pancreatic *β*-cell hypertrophy and/or hyperplasia and increased secretion of insulin. When the capacity of *β*-cells fails to compensate for the degree of insulin resistance, insulin deficiency and ultimately type 2 diabetes ensue [12]. Pancreatic *β*-cell failure displays specificity for insulin signaling pathway; while retaining capacity to respond to challenges (such as, *β*-adrenergic agonists, amino acids, and sulfonylurea drugs), cells lose their capacity to respond to glucose. If not adequately managed, hyperglycaemia may induce a glucotoxicity and lipotoxicity involving oxidative and endoplasmic reticulum stress, overexpression of proinflammatory autacoids, and increased rates of *β*-cell apoptosis [12, 13].

It is becoming increasingly evident that inflammation plays a key role in the pathogenesis of type 2 diabetes. It is well established that systemic markers of inflammation, including C-reactive protein, haemoglobin, serum amyloid A, proinflammatory cytokines and chemokines are elevated in the blood of type 2 diabetics. The source of these mediators is of multiorgan origin and, at least in part, in response to elevated concentrations of glucose and fatty acids. Both secretagogues promote proinflammatory conditions in many tissues (including pancreatic isLet-7 cells, adipose tissue, liver, and muscle), induce the release of inflammatory autacoids, and alter redox status. Chemokines further promote the recruitment of macrophages to affected tissues and together with T-cell and possibly mast cells may establish a local chronic inflammation that involves the constitutive activation of gene transcription factors such as the nuclear factor *κ*B (NF-*κ*B) family.

NF-*κ*B is a sequence-specific family of transcription factors critically involved in inflammation and innate immune responses. The NF-*κ*B family comprises at least five proteins, of which the most abundant form in unstimulated cells occurs in the cytoplasm as a heterodimer composed of two proteins, p50 and p65 bound to an inhibitory subunit, I*κ*B*α*. Upon stimulation, I*κ*B*α* is phosphorylated by an I*κ*B kinase complex, thus, targeting I*κ*B*α* for ubiquitin-dependent degradation and liberates NF-*κ*B dimers to translocate to the nucleus where they bind to the consensus sequence 5-GGGPuNNPyPyCC-3. This *κ*B motif has been identified in the promoter regions of many proinflammatory mediators, including adhesion molecules (ICAM-1), enzymes (including, inducible nitric oxide synthase, phospholipase A_2_s, cyclo-oxygenase-2, urokinase plasminogen activator, metalloproteinases, superoxide dismutase), cytokines (e.g., IL-1*β*, IL-6, TNF*α*), and chemokines (IL-8) [14].

It is noteworthy that recent studies in breast cancer [15] provide data implicating NF-*κ*B, IL-6, and Let-7 micro-RNA (miRNA) in an epigenetic switch or positive feedback loop that resets inflammatory pathways to a heightened state of responsiveness. In this model, a triggering event induces NF-*κ*B DNA binding activity and Lin28 expression and represses Let-7 miRNA action. One of the actions of Let-7 is to suppress IL6 formation. Thus, inhibition of Let-7 results in higher levels of expression of IL6 than achieved by NF-*κ*B activation alone. IL6 activation of the STAT3 transcription factor is necessary for neoplastic cellular transformation, and IL6 activates NF-*κ*B, thereby completing a positive feedback loop.

**Figure 1:**
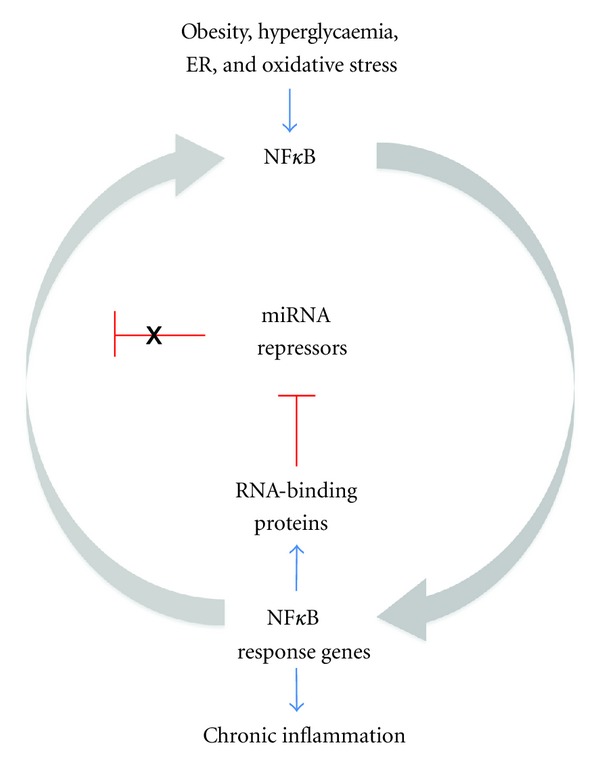
Diabetes-related triggers may initiate a positive feedback loop resulting in heighten responsiveness of the NF-*κ*B by the inhibition of miRNA repressors by NF-*κ*B-induced RNA binding proteins.

We propose that a similar mechanism may induce a sustained inflammatory response, activation of NF-*κ*B-regulated genes, and insulin resistance in type 2 and gestational diabetes. That is, that a primary pathophysiological insult (Figure 1, e.g., obesity, metabolic stress, hyperglycaemia, or hyperlipidaemia) may activate NF-*κ*B-mediated gene expression and proinflammatory regulatory pathways. Subsequent secondary or repetitive insults may induce a persistent, heightened response, the positive feedback loop being established via the induction of the RNA-binding protein Lin28 that suppresses Let-7 microRNA family members. Let-7 miRNA suppresses interleukin-6 expression (binding IL-6 mRNA though its 3′ UTR) and other growth and metabolic mediators. The resulting positive feedback loop between NF-*κ*B and, in particular, IL-6 and the inhibitory effects of Lin 28 and IL-6 on Let-7 is sufficient to result in sustained elevation and responsiveness of these pathways.

In support of this hypothesis, Zhu et al. [16] recently reported that the Lin28/Let-7 axis plays a role in the reprograming of glucose metabolism in malignancy. Lin28a and Lin28b were reported to promote insulin-sensitivity and resistance to high-fat-diet-induced diabetes. Furthermore, muscle-specific inhibition of Lin28a or overexpression of Let-7 results in insulin resistance and impaired glucose tolerance. These effects were mediated, in part, via Let-7 repression of the insulin-PI3K-mTOR pathway, including the insulin receptor, insulin receptor substrate 2, and insulin-like growth factor 1 receptor. In normal adult individuals, Lin28 expression is low and, thus, Let-7 represses the expression of a cassette of gene pathways associated with growth, cell migration, and catabolic metabolism. Under conditions where Lin28 is induced (e.g., via activation of NF-*κ*B response pathways), Let-7 repression of these genes is removed. In the case of type 2 diabetes, triggers such as obesity, oxidative stress, and inflammatory mediators may initiate aberrant activation of the NF-*κ*B pathway and initiate a feedback loop that sustains and progressively increases insulin resistance.

### 2.3. Management and Intervention

The available data support the contention that the adverse sequelae of diabetes (including microvascular, cardiovascular, and renal disease) can be, at least, ameliorated by adequate glycaemic control [17]. Recent trials have established the benefits of interventions to prevent or delay diabetes and reduce diabetes-related complications and/or associated risk factors [18–21]. Intensive lifestyle modification to promote weight loss and increase physical activity resulted in a 58% reduction in the risk of type 2 diabetes in adults with impaired glucose tolerance [21]. Early diagnosis of predisposition to type 2 diabetes and implementation of effective intervention represent a strategy to abate the incidence of type 2 diabetes and its associated health care burden.

## 3. Gestational Diabetes Mellitus

### 3.1. Epidemiology

GDM is glucose intolerance with onset or first recognition during pregnancy [22]. GDM affects ~5% of all pregnancies and its incidence is increasing in parallel with the global increase in obesity and type 2 diabetes. In the USA, GDM affects 135, 000 pregnancies per year. GDM has been associated with not only acute increased risk for complications of pregnancy but also long-term disease risks for both mother and baby (Australian Institute of Health and Welfare, 2010). Perinatal morbidity includes hyperinsulinaemia, macrosomia, hypoglynaemia, hyperbilirubinaemia, and respiratory distress syndrome which in turn may generate subsequent complications. Longer-term morbidity for the offspring includes obesity and diabetes independent of genetic factors [23–26]. GDM in the mother is associated with increased risk of overt diabetes later in life. A higher risk of developing metabolic and cardiovascular disease has been reported for women who develop GDM during pregnancy.

In 2011, the American Diabetes Association (ADA) and the International Association of Diabetes and Pregnancy Study Groups (IADPSG) revised recommendations regarding GDM. It is now recommended that patients at increased risk for type 2 diabetes be screened for diabetes using standard diagnostic criteria at their first prenatal visit. High-risk women are defined as having impaired fasting plasma glucose levels of 5.6 mmol/L to 6.9 mmol/L [100 mg/dL to 125 mg/dL]) or impaired glucose tolerance (2-hour OGTT values of 7.8 mmol/L to 11.0 mmol/L [140 mg/dL to 199 mg/dL]). Women with an HbA1c of 5.7% to 6.4% are also considered at increased risk. In these patients, confirmed fasting glucose levels of ≥7.0 mmol/L (126 mg/dL) or random glucose levels ≥11.1 mmol/L (200 mg/dL) are also diagnostic of diabetes. The ADA and the IADPSG recommended that such high-risk women with diabetes diagnosed on the basis of standard diagnostic criteria receive a diagnosis of overt rather than gestational diabetes.

At 24 to 28 weeks of gestation, all women not known to have diabetes (including high-risk women if the initial testing was normal) should undergo a 75 g OGTT, with diagnosis of GDM based upon the finding of 1 abnormality, rather than the previously recommended 2.

### 3.2. Aetiology

Normal pregnancy is attended by significant changes in maternal metabolism [27] that are induced, at least in part, by the release of placenta-derived autacoids [28–30]. Early pregnancy is anabolic and associated with the accretion of maternal fat. Late pregnancy is catabolic and characterised by increasing insulin resistance, lipolysis, hyperinsulinaemia, hyperglycaemia, increased postprandial fatty acid concentrations, and declining maternal fat reserves. The net effect of these late gestation changes are increased availability of energy and anabolic substrates to sustain the growth of the feto-placental unit, increased utilisation of glycolytic energy production, and increased free fatty acid availability [31]. Late pregnancy is also associated with a decreased ability to produce glucose via gluconeogenesis, glycogenolysis and lipolysis. This is in part a consequence of an attenuation of hypoglycaemia to induce glucagon, norepinephrine and cortisol.

It has been suggested that GDM and type 2 diabetes may share common pathogenic mechanisms, however, pregnancy may serve to unmask disease in those women who are predisposed and destined to develop type 2 diabetes later in life. Similar to type 2 diabetes, GDM is manifested by the inability of pancreatic *β*-cell insulin release to compensate for pregnancy-induced insulin resistance resulting in maternal hyperglycaemia and hyperinsulinaemia. Moderate hyperinsulinaemia is considered adaptive during normal pregnancy and a response to increased energy utilisation and demand by the developing fetus. In GDM, the adaptive changes in insulin resistance extend beyond those normally observed [32, 33]. For example, during normal pregnancy, insulin-stimulated glucose transport by skeletal muscle fibers is reduced by ~40%. In women with GDM, glucose transport has been reported to be reduced by up to 65% [34]. Environmental (modifiable) risk factors, including, preconception conditioning, maternal diet and exercise, and other lifestyle factors, may impact on the severity of its manifestation. In most cases, however, symptoms of metabolic dysfunction disappear postpartum following the withdrawal of placental autacoid mediators.

The effects of hyperglycaemia on pregnancy outcome are underpinned by experimental studies that identify putative effector pathways by which exposure to high glucose concentrations may alter placental and maternal adipose tissue phenotype and responsiveness [35–37]. Similar to type 2 diabetes, the role of inflammatory mechanisms in disease progression is evident. Increased biomarkers of oxygen radical damage and an impairment of antioxidant defense have been identified in individuals with type 2 diabetes [38] and in women with GDM [37, 39–41]. Previously, we demonstrated that the placentae of women with GDM display a reduced capacity to respond to oxidative stress in terms of 8-isoprostane and tumour necrosis factor *α* (TNF *α*) release [35]. We concluded that GDM placenta may be preconditioned by transient intracellular oxidative stress. The role of oxidative stress in the aetiology of GDM has recently been reviewed [42].

## 4. Screening for Diabetes

### 4.1. Screening for GDM

**Figure 2:**
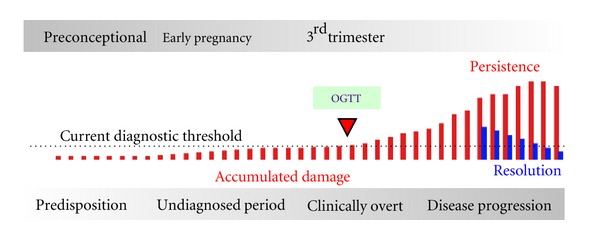
GDM disease progression. GDM is currently diagnosed (Clinically Overt) in 3rd trimester (24–28 weeks of gestation) following an oral glucose tolerance test (OGTT). Using this diagnostic threshold, there is no opportunity to prevent pathological changes (accumulated damage) that may occur during 1st and 2nd trimester (Undiagnosed Period). The implementation of screening tests during early pregnancy or the preconception period affords opportunity to identify women at risk of disease and to evaluate intervention strategies on pregnancy outcome and the long-term health of both mother and baby.

Currently, GDM is diagnosed in the late second or early third trimester of pregnancy. Any pathology is probably already established by this time and reversal of the potential adverse perinatal outcomes may be limited. The lack of a reliable early test for GDM has hampered the development of useful intervention therapies that may impact not only on the acute but long-term health outcomes (Figure 2). Thus, there is a need to diagnose and predict GDM earlier so that appropriate management can be initiated and tailored to the needs of the patient in order to minimise perinatal complications and their sequelae.

GDM is currently diagnosed by an In Vitro Diagnostic (IVD) Oral Glucose Tolerance Test performed at 24–28 weeks of gestation. A glucose load (75 g) is administered to fasting individuals, and blood glucose concentration is determined at 1 hour and 2 hours [43, 44]. The Third International Workshop-Conference on GDM emphasised the critical importance of developing new diagnostic criteria that are based on the potential to detect pregnancies at risk for adverse perinatal outcome as a result of maternal hyperglycaemia, rather than placing primary emphasis on the identification of mothers at risk for progression to diabetes outside of pregnancy. With the obesity epidemic well entrenched in the Western world and with more women delaying pregnancy and the associated increase in pre-pregnancy body mass index (BMI), the incidence of GDM is increasing irrespective of the diagnostic criteria used.

First trimester pregnancy and preconceptional risk-factors for GDM have been identified including family history of GDM and/or diabetes [45], maternal pregnancy weight gain [46, 47], fasting plasma glucose [48], 1-hour glucose challenge test [49], oral glucose tolerance test [50], and haemoglobin A1c [51] adiponectin [52, 53], C-reactive protein [54], serum triglycerides [55], sex hormone-binding globulin [56], placental growth factor [57] leptin [58], oxidised DNA [59], and follistatin-like-3 levels [60]. Although some have been able to provide a good negative predictive measure for subsequent GDM [61], most tests suffer from poor positive predictive values and are of limited efficacy.

It is now widely acknowledged that single biomarkers are unlikely to deliver significant incremental gain in sensitivity and specificity that will be required for the development of effective screening and classification tests requisite for the implementation of personalised medicine. New approaches based upon the measurement of multiple biomarkers of disease risk afford opportunity to increase diagnostic test sensitivity and specificity. Even the use of two biomarkers can deliver improved performance [62]. The use of modelling algorithms to combine multiple known biomarkers (e.g., candidate-based approaches) similarly may increase diagnostic efficiency and deliver classification models of clinical utility [63–66]. Both candidate-based applications (i.e., in which the identity of the analytes being measured are well-established [67]) and signature profiling applications (i.e., in which characteristic patterns or motifs within a signal profile are identified, see [68]) may be utilised in the development of multivariate modelling strategies for the delivery of more informative diagnostic tests [69].

A recent trend in the development of more efficient diagnostic tests has been the use of algorithm-based multivariate index assays (IVDMIAs). With the development of this new class of IVD, the discipline has sought new biostatistical approaches for assessing and quantifying incremental gains in diagnostic efficiency. Traditionally, the area under the receiver operator characteristic curve (AUC) has been used as a measure and comparator of diagnostic efficiency. Several investigators have argued that this measure alone may be imperfect and inefficient for comparing the true clinical usefulness of alternative marker panels [70, 71]. These authors reviewed several biomarker studies and observed that when evaluating improvement in risk assignment of biomarkers, very large odds ratios were often associated with very small increases in the AUC. This feature of the receiver operator characteristic curve analysis limits its utility in identifying putative beneficial contributions of new biomarkers to algorithm-based models. Pencina et al. [72] therefore, proposed the use of two new methods for evaluating the diagnostic efficiency of biomarkers. These two methods are (i) Net Reclassification Improvement (NRI) and (ii) Integrated Discrimination Improvement (IDI). The NRI is based on counts of the number of true positives showing an increase in probability of an event and the number of true negatives showing a decrease in probability of an event. The IDI is based on the integral of sensitivity and specificity of all possible thresholds. These new biostatistical approaches may facilitate the development of biomarker panels with improved diagnostic efficiency and aid in the screening and earlier detection of diabetic conditions.

IVDMIA approaches are being developed for risk assignment modalities for use in the first trimester of pregnancy, including the evaluation of multiple candidate-based profiling of blood-borne biomarkers. For example, we measured multiple plasma biomarkers at 11 weeks of gestation in women who subsequently experienced a normal pregnancy outcome and women who subsequently developed gestational diabetes [73]. Of the biomarkers considered, algorithms that included adiponectin, insulin, and random blood glucose delivered the greatest diagnostic efficiency when compared to individual biomarkers alone. The IVDMIA increased AUC by more than 10%. This simple example demonstrates the putative benefit of a multimarker approach for improving diagnostic efficiency.

### 4.2. Screening for Type 2 Diabetes after GDM

As discussed above, GDM may unmask a predisposition to type 2 diabetes and, as such, GDM may be diagnostic for type 2 diabetes. A recent meta-analysis [74] reviewed 20 studies conducted between 1960–2009 to estimate the relative risk of developing type 2 diabetes following GDM. The combined cohort involved more than 675,000 pregnancies of which 31,867 cases of GDM were identified (i.e., 4.7% of all pregnancies included in the analysis). Of these cases of GDM, 10,859 incident cases of type 2 diabetes were identified. The relative risk for type 2 diabetes following GDM was estimated to be 7.43 (compared to women who had normoglycaemic pregnancies). Bellamy et al. [74] suggested that increased awareness of the risk of type 2 diabetes after GDM could provide an opportunity to test and use dietary, lifestyle, and pharmacological interventions that might prevent or delay the onset of type 2 diabetes.

While the conclusion that GDM is a risk factor for type 2 diabetes is supported by the available data, it is pertinent to note that more than 95% of women in the cohort did not develop gestational diabetes and that more that 64% of women who have had GDM do not have type 2 diabetes 20 years postindex pregnancy. The prevalence of type 2 diabetes in women 10–20 years after index pregnancy is estimated to be ~5%; thus, in a cohort of 675,000, ~33,700 incidence cases would be expected, that is, 22,841 cases in this cohort were not associated with a previous GDM pregnancy (i.e., a false positive rate of 0.659). The positive predictive value of GDM as a diagnostic test for type 2 diabetes is only 34% (sensitivity = 0.322 and specificity of 0.967).

Similarly, Göbl et al. [75] recently reported results of a small prospective cohort (*n* = 110) with 10-year followup of women who experienced GDM in which 78.7% of women did not develop type 2 diabetes. Of the 21.3% who did progress to type 2 diabetes, a multivariate analysis identified 2-hour oral GTT concentration, HDL cholesterol, and age as the best predictors. Women with two or more risk factors were at a higher risk than women with only one.

It has further been proposed that women with a previous GDM pregnancy should be followed up by OGTT postpartum and, if positive, intervention and monitoring strategies implemented. While this may be appropriate, it does not assist the ~68% of women who develop type 2 diabetes in the absence of GDM complicated pregnancies. Specifically targeting and monitoring women with a previous GDM pregnancy is an aid in identifying predisposition to type 2 diabetes but is a poor stand-alone diagnostic test for type 2 diabetes in women. Alternative strategies for early detection screening, intervention, and prevention are requisite to reducing the overall burden of type 2 diabetes at a community level.

The objectives of any disease screening program are to

1. identify asymptomatic individuals at higher risk of, or predisposition to disease,
2. afford the opportunity for treatment or prevention of disease thus limiting severity,
3. reduce disease burden in the community.

A number of opportunities for developing screening applications that target women of reproductive age exist, including the development of preconception screening.

### 4.3. Preconception Conditioning and Screening

The concept that information defining contemporary environmental conditions is coded in maternal physiology and is sensed and informs and adapts the development of the fetus is not novel [76, 77]. This tenet is the basis of epigenetics and the developmental origins of adult disease [78]. Modifiable maternal and environmental factors reported to affect pregnancy outcome and/or disease risk in the offspring include BMI [79, 80], weight gain during pregnancy [47], diet [81–83] physical activity [84, 85], preexisting diabetes [86, 87], and alcohol consumption [88]. The placenta functions as an environmental sensor for the embryo and not only integrates information encoded within the maternal *milieu* but its own ontogenic development and function may be altered by such information. Known modifiers of the placental epigenome in human and animal models include micronutrients [89, 90], diet [91], smoking [92], bacterial infection [93], obesity [94], stress [95], diabetes [96], and hypertension [97, 98].

Based upon the available data, a sound case can be made for the implementation and evaluation of preconception care programs that attempt to optimise or, at least, improve general health, life style, and conditioning of women and their partners before conception. The premise underpinning promoting preconception in addition to prenatal care is that for some maternal conditions and exposures, altered programing and/or damage can occur before prenatal care begins. To promote normal placentation and reduce risks of complications of pregnancy, education and appropriate interventions must be identified and implemented prior to conception.

In 2006, the Centers for Disease Control and Prevention (CDC) published a report of the CDC/ATSDR Preconception Care Work Group and the Select Panel on Preconception Care [99, 100]. The panel identified four primary objectives:

1. improve knowledge, attitudes, and behaviors of men and women related to preconception health,
2. assure that all women of childbearing age receive preconception care services that will enable them to enter pregnancy in optimal health,
3. reduce risks identified by a previous poor pregnancy outcome through interventions during the interconception period, which can prevent or minimize health problems for a mother and her future children,
4. reduce disparities in adverse pregnancy outcomes.

The actual benefits of preconception care programs remain to be established as there is a paucity of randomised control data on the health outcomes and economic benefit of attempts to improve preconception maternal (and paternal) health. Several studies, however, have reported some evidence of positive financial returns for preconception counseling for women with diabetes, based on savings in hospitalisation costs [101, 102]. Similarly, case control studies on the health care costs associated with maternal obesity provide further support, reporting that the cost of prenatal care was 5 times higher in mothers who were overweight before pregnancy than in normal-weight control women [103]. More recently, Moos and Bennett [104] reviewed the evidence supporting preconception care for diabetic women.

The implementation of preconception care programs afford opportunity for more broadly based screening for prediabetic conditions and early intervention to reduce the incidence of type 2 diabetes. The efficacy of screening this cohort has yet to be established it would, however, afford opportunity for longitudinal monitoring of biomarkers. Longitudinal monitoring is an approach that has proved effective in increasing the diagnostic performance of oncology diagnostics [105, 106]. It is likely that such an approach would also improve the positive predictive value for the diagnosis of type 2 diabetes.

## 5. Conclusion

Type 2 diabetes is a *“communicable disease” *that is transmitted between individuals and intergenerationally by the adoption of societal and lifestyle behaviors—behaviours that challenge fundamental energy homeostasis. The juxtaposition of a susceptible genetic background with the ability to access a surfeit of energy dense foods without counterpoise energy expenditure predisposes to obesity and failure of glycaemic control. Modifiable risk factors have been identified that may reduce disease severity. Early identification of individuals at higher risk of developing type 2 diabetes will play a critical role in improving disease management and health outcomes. Of significant promise is the development and implementation of high performance IVDMIAs and their use in longitudinal monitoring programs.

## Abbreviation

IVDMIA: In Vitro Diagnostic Multivariate Index Assay.
